# High-Performance Polyimides with Enhanced Solubility and Thermal Stability for Biomimetic Structures in Extreme Environment

**DOI:** 10.3390/biomimetics11010061

**Published:** 2026-01-12

**Authors:** Jichao Chen, Jiping Yang, Zhiyong Ma, Zhijian Wang, Yizhuo Gu

**Affiliations:** 1 Tianmushan Laboratory, Hangzhou 310023, China; bhtb028@tmslab.cn; 2 Key Laboratory of Aerospace Advanced Materials and Performance, Ministry of Education, School of Materials Science and Engineering, Beihang University, Beijing 100191, China; 3 Beijing Advanced Innovation Center for Soft Matter Science and Engineering, State Key Laboratory of Organic-Inorganic Composites, College of Chemical Engineering, Beijing University of Chemical Technology, Beijing 100029, China

**Keywords:** polyimide, siloxyne–alkyne diamine, thermal stability, DFT calculation, structure–property relationship

## Abstract

Designing the high-performance polyimides (PIs) for the biomimetic structures, which are used in extreme conditions, remains greatly challenging, due to the conflict between processability and thermal stability. Here, we report a series of silicon–alkyne-functionalized diamine-based polyimides that exhibit remarkable processability and thermal stability. The incorporation of bulky siloxy groups disrupts chain packing and increases free volume, enabling excellent solubility in polar solvents, while the rigid fluorene core enhances chain stiffness. DFT calculations confirm twisted molecular geometries (Si bond angle ≈ 103°, dihedral angle ≈ 89°) which weak π–π stacking, while heterogeneous electrostatic potentials enable favorable noncovalent interactions (e.g., C–F···H–C), promoting solvent diffusion. After thermal curing, the obtained product shows a high decomposition temperature (*T*_d5%_ = 560 °C), char yield of 72.0% at 800 °C, and glass transition temperature (*T*_g_) of 354.6 °C. Meanwhile, locally planar fluorene units retain inherent thermal stabilization benefits through constrained rotational mobility. These results demonstrate a spatially decoupled siloxy–alkyne design that synergistically enhances molecular flexibility, disorder, and electronic stability, offering a molecular strategy for tailoring PI-based matrices to meet the demands of emerging biomimetic architectures and other high-performance composites operating under severe thermal loads.

## 1. Introduction

**Engineering** applications greatly benefit from biomimetic structures inspired by the delicate, flexible mechanics found in nature. However, implementing such architectures in extreme thermal or chemically aggressive environments requires matrix polymers that can maintain structural integrity while providing high thermal stability and processability requirements that conventional resins often fail to meet. Polyimides have already been employed as matrix or interphase resins in nacre-inspired layered composites, bioinspired honeycomb and lattice structures, and thermal-protection skins, owing to their excellent heat resistance and solution processability [1,2,3,4]. These applications highlight the need for continued advances in polyimide molecular design to address the longstanding trade-off between thermal robustness and processability. The continuing demand for materials capable of withstanding extreme environments is thus driving the pursuit of further improvements in the thermal properties of PI resins. Incorporating silicon into the PI backbone is an effective strategy for improving thermal-oxidative resistance and increasing char yield at elevated temperatures, owing to the formation of thermally stable Si–O bonds and silica-like inorganic networks during decomposition [5,6,7,8]. The incorporation of silicon-containing moieties, such as siloxane groups, has emerged as a promising approach to overcoming the inherent limitations of conventional aromatic PIs. The incorporation of siloxane units imparts enhanced thermal stability and flexibility to the polymer matrix, attributable to the strong Si–O bonds and the capacity of siloxane chains to act as effective thermal barriers [9,10,11,12,13]. Kuo et al. [14] developed a thermally stable silicone-modified resin by introducing cage-shaped polyhedral oligomeric silsesquioxane (POSS) into a dianhydride, thereby generating a novel dianhydride monomer that was subsequently grafted onto a polybenzimidazole resin. This resin represents a promising candidate for ablation-resistant applications, owing to its high char yield (76%) and elevated thermal decomposition temperature (521 °C). Modifications employing silicon-containing diamine monomers are more prevalent than those utilizing silicon-containing dianhydride counterparts. Liu et al. [15] developed a phenylethynyl-terminated polyimide oligomer derived from bis(*p*-aminophenoxy)tetramethyldisiloxane (APTMDS) and milled carbon fibers. During selective laser sintering (SLS) and post-curing, the oligomer undergoes phenyl crosslinking reactions and oxidative crosslinking of siloxane units, resulting in polyimide components exhibiting a tensile strength of 82 MPa and a glass transition temperature of 419 °C. The honeycomb structures fabricated using this strategy exhibit higher specific compressive strength and can withstand temperatures up to 400 °C. APTMDS was also utilized by Fan et al. [10] to prepare a series of thermoset siloxane-containing polyimide resins exhibiting excellent processability and heat resistance. The modified siloxane-containing oligopolyimides demonstrated relatively high glass transition temperatures (*T*_g_ = 345.3 °C). The enhanced thermal stability stems from the oxidative crosslinking of siloxane groups during curing, in addition to phenylacetylene crosslinking. This process leads to the in situ formation of an inorganic silica-like structure interconnected by organic polyimide segments. This organic/inorganic hybrid nature confers the polyimide resin with improved thermal properties. Liu et al. [16] synthesized novel siloxane-type diamines, which were uniformly incorporated into the polyimide main chain, thereby imparting exceptional thermal stability (*T*_d5%_ of 458 °C).

In parallel, the incorporation of alkynyl moieties has emerged as a promising strategy to achieve enhanced crosslinking density and superior thermo-oxidative stability, particularly under high-temperature oxidative environments, owing to their unique electronic and steric effects [17,18,19]. Recently, increasing attention has been directed toward combining siloxane and acetylene groups within a single polymer architecture to synergistically enhance the properties of polyimide resins. For instance, Fan et al. [20] reported the synthesis and thermal curing of a novel polyhedral oligomeric octa(propargylaminophenyl) silsesquioxane, which exhibited enhanced thermal stability and mechanical properties compared with conventional polyimides. Moreover, Hu et al. [21] developed a new thermoset polyimide resin (SiOPI) containing silicon and alkynyl structures in the main chain, synthesized using 4,4′-oxybis(phthalic anhydride) (ODPA) as the dianhydride monomer and the corresponding diamine. The incorporation of silyl groups into the PI backbone enhances resin flexibility and improves solubility and processability. By virtue of the alkyne groups, the cured polyimide forms dense reticulated structures analogous to benzene rings, thereby imparting excellent heat resistance and mechanical properties (*T*_g_ = 367 °C). These studies highlight the potential of integrating siloxane and acetylene groups to develop advanced polyimide materials with superior performance. However, most existing studies have focused on the direct connection between siloxane and acetylene groups, which may hinder the optimization of their respective contributions. Direct integration of Si–O and alkynyl units within the same molecular backbone often results in undesired steric interactions and processing incompatibilities.

Recent studies have explored spatially separated designs, in which Si–O and alkynyl functionalities are incorporated into the molecular architecture without direct bonding. This decoupled approach provides enhanced structural tunability, crosslinking control, and phase stability. It has been reported to significantly delay the onset of thermal decomposition compared with conventional systems [11]. Despite this progress, a systematic understanding of the structure-property-performance correlation remains underdeveloped, particularly with respect to molecular design principles and network formation behaviors. Such decoupled architectures enable independent modulation of thermal flexibility (via Si–O segments) and crosslinking density (via alkyne moieties), thereby overcoming the long-standing trade-off between processability and heat resistance. Notably, the absence of direct Si–C≡C linkages mitigates premature thermal degradation and broadens the design space for spatial network formation during thermal curing. Compared with direct covalent linkage between siloxane (Si–O) and alkynyl groups, spatial separation of these functionalities within the molecular architecture provides distinct chemical and material advantages. From a chemical perspective, decoupling siloxane and alkynyl units minimizes undesired side reactions, including premature oxidative crosslinking and steric hindrance during synthesis and thermal treatment. This spatially segregated design enables independent optimization of chain flexibility and crosslinking density, thereby improving structural tunability. From a materials standpoint, this arrangement allows flexible Si–O segments to impart thermal relaxation and processability, whereas alkynyl groups contribute to high crosslinking density and oxidative resistance. The resulting hybrid resins exhibit superior thermal stability, enhanced film-forming ability, and improved compatibility with high-temperature applications. Therefore, the decoupled siloxane–alkynyl architecture constitutes a promising strategy for designing advanced polyimide systems with multifunctional performance under extreme conditions.

**Scheme 1:**
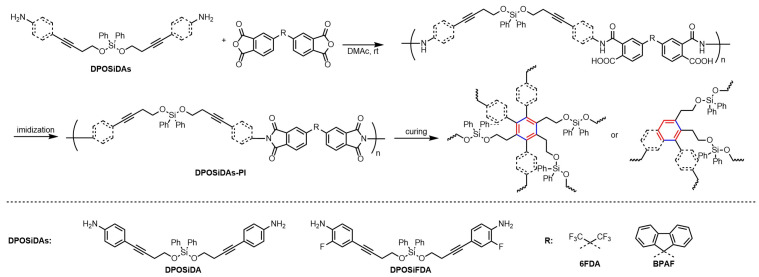
Schematic illustration of the preparation of the siloxyalkynylamines-based polyimide (DPOSiDAs-PI).

Extreme-environment biomimetic structures, including nacre-like composites, architected lattices, and thermal-protection aerogels, depend on matrix polymers capable of enduring severe thermal conditions. High-performance polyimides offering both exceptional thermal stability and good processability thus provide a compelling materials basis for advancing these bioinspired architectures. In this work, we propose a spatially decoupled siloxy–alkyne diamine design to independently regulate chain mobility and crosslinking capability. Three polyimides derived from novel diamines (Scheme 1) were systematically synthesized. Then, the dianhydride monomer in an equimolar ratio to the diamine monomer was added to undertake copolymerization, resulting in a poly(amic acid) (PAA) solution with a solid content of 20 wt%. The resin is dried in the vacuum oven and cured in an air atmosphere at elevated temperature to produce dark red samples. The PI resin exhibits excellent solubility, while the cured product shows outstanding thermal stability, and high char yields, but also enable a clear structure-performance relationship that highlights the cooperative contribution of siloxy flexibility and alkyne-derived crosslinking. Their unique combination of solution processability and exceptional thermal robustness-further elucidated through DFT-assisted conformational and electronic analyses-provides a materials platform well suited for the fabrication of high-performance biomimetic structures, thermally resilient composites, and architected materials for extreme-environment applications.

## 2. Materials and Methods

### 2.1. Materials

All reagents and solvents were provided by Energy Chemical (Shanghai, China) and Shanghai Titan Scientific Co., Ltd. (Shanghai China), respectively. They were used without further purification. The progress of the reaction was followed by thin-layer chromatography (TLC) on commercial silica gel plates, with visualization under 254 nm UV light. Purification by flash chromatography was carried out on silica gel, eluting with a petroleum ether (PE)/ethyl acetate (EtOAc) gradient.

### 2.2. Synthesis of Siloxyalkynylamines

**Scheme 2:**
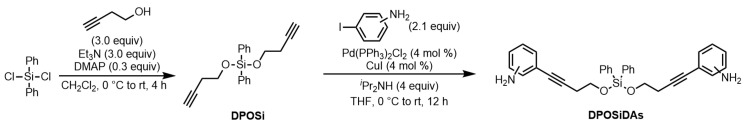
Synthesis of DPOSiDAs.

To a solution of 3-butyn-1-ol (30 mmol, 3.0 equiv), 4-dimethylaminopyridine (DMAP, 3 mmol, 0.3 equiv), and triethylamine (Et_3_N, 30 mmol, 3.0 equiv) in dry CH_2_Cl_2_ (20 mL), dichlorodiphenylsilane (10 mmol, 1.0 equiv) was added at 0 °C dropwise under an argon atmosphere. The reaction mixture was stirred at room temperature for 4 h. After completion (as indicated by TLC), the mixture was quenched with H_2_O and extracted with CH_2_Cl_2_. The organic extracts were combined, washed with brine, dried over anhydrous MgSO_4_, and evaporated under reduced pressure. The crude residue was purified by silica-gel flash chromatography (eluent: PE/EtOAc = 50:1 to 25:1) to afford DPOSi (see Scheme 2).

To an oven-dried round-bottom flask were added DPOSi (5 mmol, 1.0 equiv), iodoaniline derivatives (10.5 mmol, 2.1 equiv), Pd(PPh_3_)_2_Cl_2_ (0.2 mmol, 4 mol %), and CuI (0.2 mmol, 4 mol %) in anhydrous THF (25 mL, 0.2 M). And then diisopropylamine (*^i^*Pr_2_NH, 20 mmol, 4.0 equiv) was added under an argon atmosphere at 0 °C. The mixture was stirred at room temperature for 12 h until the DPOSi was completely consumed, as monitored by TLC. Then, the reaction was quenched with saturated NH_4_Cl solution and extracted with EtOAc. The combined organic phases were washed with brine and dried over anhydrous MgSO_4_. After filtration and solvent removal under reduced pressure, the crude product was purified by silica-gel flash chromatography (PE/EtOAc) to afford DPOSiDA and DPOSiFDA.

**4,4′-(((diphenylsilanediyl)bis(oxy))bis(but-1-yne-4,1-diyl))dianiline (DPOSiDA)**. It was purified by column chromatography on silica gel (PE/EtOAc = 4:1 to 1.5:1) to afford the product as a yellow oil; **^1^H NMR (400 MHz, DMSO)** (Figure S1)δ 7.70–7.67 (m, 4H), 7.49–7.44 (m, 2H), 7.40–7.37 (m, 4H), 7.06–7.03 (m, 4H), 6.52–6.49 (m, 4H), 5.39 (s, 4H), 3.93 (t, *J* = 6.6 Hz, 4H), 2.67 (t, *J* = 6.6 Hz, 4H); **^13^C NMR (100 MHz, DMSO)** (Figure S2) δ 149.2, 135.1, 132.8, 132.7, 131.1, 128.5, 114.1, 109.8, 84.3, 83.3, 62.2, 23.5.

**4,4′-(((diphenylsilanediyl)bis(oxy))bis(but-1-yne-4,1-diyl))bis(2-fluoroaniline) (DPOSiFDA)**. It was purified by column chromatography on silica gel (PE/EtOAc = 4:1 to 1.5:1) to afford the product as a yellow oil; **^1^H NMR (600 MHz, DMSO)** (Figure S3) δ 7.67 (d, *J* = 7.4 Hz, 4H), 7.47 (t, *J* = 7.5 Hz, 2H), 7.39 (t, *J* = 7.2 Hz, 4H), 6.95 (d, *J* = 12.2 Hz, 2H), 6.89 (d, *J* = 8.2 Hz, 2H), 6.67 (t, *J* = 8.8 Hz, 2H), 5.44 (s, 4H), 3.92 (t, *J* = 6.5 Hz, 4H), 2.67 (t, *J* = 6.5 Hz, 4H); **^13^C NMR (150 MHz, DMSO)** (Figure S4) δ 150.0 (d, *J* = 237.6 Hz), 137.5 (d, *J* = 13.0 Hz), 135.0, 132.6, 131.1, 128.7, 128.5, 118.1 (d, *J* = 19.6 Hz), 116.3 (d, *J* = 5.3 Hz), 110.0, 85.2, 62.0, 23.3; **^19^F NMR (376 MHz, DMSO)** (Figure S5) δ -134.8.

### 2.3. Synthesis and Preparation of Siloxyalkynylamines-Based Polyimide (DPOSiDAs-PI) Resin

A series of polyimides was synthesized by adjusting the structure of siloxyalkynylamines and the kind of dianhydride using a conventional two-step process (Scheme 1). Taking the synthesis of DPOSiDA/BPAF polyimide as an example, the process is as follows: DPOSiDA (5.0 mmol) was dissolved in DMAc (10 mL) under an argon atmosphere and stirred at room temperature for 1 h. Subsequently, BPAF (5.0 mmol) dissolved in DMAc (9 mL) was added dropwise to the premade diamine solution. The mixture was stirred at room temperature for 12 h, resulting in a poly(amic acid) (PAA) solution with a solid content of approximately 20%. Acetic anhydride (10 mmol) and triethylamine (10 mmol) were then added, and the reaction was continued for 5 h at 60 °C. Upon cooling to ambient temperature, the reaction mixture was transferred into ethanol to induce precipitation. The crude solid was isolated by filtration, rinsed first with ethanol and then with deionized water, and finally dried under reduced pressure at 100 °C to constant mass.

### 2.4. Preparation of DPOSiDAs-Based Polyimide Film

The aforesaid solution was copolymerized for approximately 12 h by adding the dianhydride monomer in an equimolar ratio to the diamine monomer, resulting in a poly(amic acid) (PAA) solution with a solid content of 20 wt%. Prior to curing, entrapped air bubbles were removed by allowing the resin to stand at room temperature for several minutes. The resin was dried in a vacuum oven at 80 °C for eight hours, then heated to 100 °C and left for four hours. After that, the glass plate was dried in an air atmosphere at 160 °C, 200 °C, 250 °C, and 300 °C for each two-hour interval to produce the membranes and dark red resin samples. Representative photographs of the polyimide powders before and after the stepwise curing are provided in Figure S6 as basic visual evidence of sample appearance and macroscopic uniformity.

### 2.5. Characterization

^1^H NMR, ^13^C NMR and ^19^F NMR spectra were recorded with a Bruker AVQ-400/600 spectrometer instrument in DMSO-*d*_6_. FT-IR was conducted using a Thermo Fisher Scientific Nicolet iS20 spectrometer. The tanδ–T curves of the membranes were tested by a Dynamic Mechanical Analyzer (DMA) TA Q8000 with a heating rate of 5 °C/min and a frequency of 1 Hz in the range from 30 °C to 450 °C under N_2_ environment. According to the position of the maximum tanδ peak, the glass transition temperatures (*T*_g_) were determined. Thermo-decomposed temperatures were assessed using a TG209F1 analyzer under a continuous flow of nitrogen or air, with a heating rate of 10 °C/min. All TGA measurements were performed in triplicate (n = 3), and the results are presented as mean ± standard deviation.

### 2.6. Density Functional Theory (DFT) Calculation

DFT calculations were performed using the Gaussian 16 program. The ground-state structures were geometry-optimized using the B3LYP hybrid functional in conjunction with the 6-31G(d,p) basis set. The electrostatic potential (ESP) was evaluated by Multiwfn [22,23]. The calculations mainly investigate the molecular conformation and electronic charge distribution of the repeating segments, whereas the actual polymer configurations and their relevance to material applications are considerably more complex.

## 3. Results

### 3.1. Structural Characterization of DPOSiDAs-Based Polyimide

**Figure 1:**
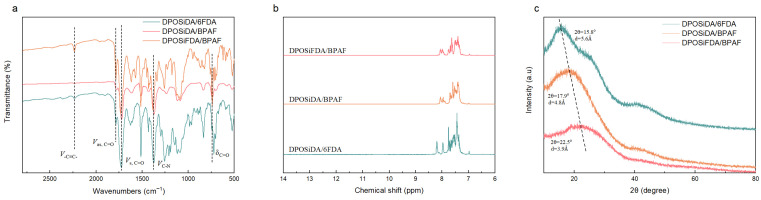
Structural characterization of different DPOSiDAs-PI resins: (**a**) FT-IR spectra, (**b**) ^1^H NMR spectra, and (**c**) XRD patterns.

Figure 1 presents the FT-IR spectra, ^1^H NMR spectra, and XRD patterns of three DPOSiDAs-based polyimide resins (DPOSiDA/6FDA, DPOSiDA/BPAF, and DPOSiFDA/BPAF). As shown in Figure 1a, the asymmetric and symmetric stretching vibrations of the imide carbonyl (C=O) groups appear at 1776–1785 cm^−1^ and 1722–1731 cm^−1^, respectively. Characteristic absorptions of the C–N stretching (1369–1380 cm^−1^) and C=O bending (721–737 cm^−1^) modes further confirm the successful synthesis of the polyimide structure [24]. The C≡C stretching band at 2235 cm^−1^ and the Si–O–C bending/stretching band at 1082–1087 cm^−1^ were clearly observed in the uncured samples. The disappearance of the C≡C band after two hours of thermal curing indicates complete crosslinking of the prepolymer (Figures S7–S9). The ^1^H NMR spectra of the DPOSiDAs-PI resins in DMSO-*d*_6_ are shown in Figure 1b. Obviously, no –COOH resonance at ~13 ppm or –NH resonance at 10–11 ppm was detected, confirming complete imidization of the poly(amic acid) to polyimide and successful synthesis of the target resins [25]. These FT-IR and ^1^H NMR results collectively verify the successful synthesis of the DPOSiDAs-PI resins. The molecular packing of the resins was further investigated by XRD, as shown in Figure 1c. All DPOSiDAs-PI resins exhibit broad diffuse diffraction peaks (2θ ≈ 10–35°), indicating their amorphous nature and absence of long-range crystallinity. The bulky phenyl substituents and asymmetric backbone geometry hinder regular crystallization by weakening intermolecular interactions. The non-planar stereostructure induced by sp^3^-hybridized silicon atoms further disrupts chain stacking. The introduction of silicon-bonded diphenyl groups increases free volume and further reduces crystallinity. By comparing the distances between molecular chains using the Bragg equation (nλ = 2dsinθ), the order of intermolecular spacing was found to be: DPOSiFDA/BPAF (3.9 Å) < DPOSiDA/BPAF (4.8 Å) < DPOSiDA/6FDA (5.6 Å). The bulky and rigid fluorenyl core of BPAF, along with its two phthalic anhydride substituents, introduces pronounced steric hindrance. These features impede regular stacking of polymer segments, causing reduced interchain spacing, thereby broadening the XRD peaks. Structurally, fluorination weakens intermolecular forces, enlarges interchain spacing, and decreases packing density. These structural modifications reduce crystallinity and broaden the XRD peaks. Moreover, fluorine atoms can induce chain twisting, further disrupting ordered packing. Collectively, these effects account for the broadening of XRD peaks upon fluorination. Substitution of 6FDA with BPAF lowers interplanar spacing (d) and shifts peaks to higher 2θ values, owing to distorted conformations and the rigid fluorene core that promotes more compact stacking. Weak C–F···H–C and C–F···π interactions, together with strong C–F–induced polarization, enhance interchain cohesive energy, shifting peaks to higher 2θ values.

### 3.2. Solubility and Thermal Properties

The room-temperature solubility of DPOSiDAs-PI resins in typical aprotic polar solvents (DMAc, DMF, NMP, DMSO, THF, and toluene) is summarized in Table 1. The addition of bulky siloxy groups disrupts chain packing and increases free volume between polymer chains. This facilitates solvent penetration, thereby enhancing solubility in organic media. Consequently, the resins readily dissolve at room temperature in DMAc, DMF, NMP, and DMSO. The prepolymer also shows partial solubility in the less polar solvent toluene. Moreover, the incorporation of flexible segments reduces chain rigidity and lowers the rotational energy barrier. This weakens intermolecular interactions, thereby improving solubility. The non-coplanar twisted conformation of the 9,9-bis(phenylene)-substituted fluorene nucleus in BPAF significantly weakens π–π stacking and crystallinity. Compared with 6FDA, BPAF-based resins show superior solubility due to polar carbonyl groups, which enhance dipole–dipole interactions with solvents and synergistically increase free volume and solvent diffusivity. Furthermore, mild fluorination enhances solubility in polar solvents by reducing dispersion forces, increasing free volume, and reinforcing C–F···H–C interactions.

**Figure 2:**
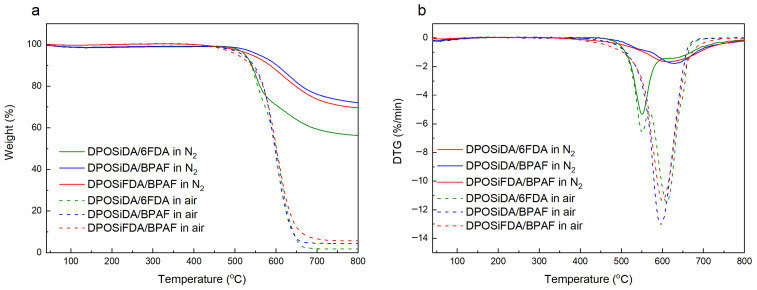
TGA and DTG curves of DPOSiDAs-based polyimides in N_2_ and air.

Thermogravimetric analysis (TGA) was conducted to evaluate the thermal stability of the DPOSiDAs-based polyimides. Figure 2 shows the TG and DTG curves, with detailed thermal data summarized in Table 2. All three resins exhibited excellent thermal stability. The *T*_d5%_ of the 6FDA-based resin reached 525 °C, yet it exhibited the lowest thermal resistance among the samples. In contrast, the BPAF-based system exhibited a maximum *T*_d5%_ of 560 °C. Thermal curing generated a rigid crosslinked network enriched with unsaturated alkynyl groups, thereby enhancing thermal stability [26]. Similarly, 6FDA-based resins showed higher weight loss with 56.0% residue at 800 °C, whereas BPAF-based resins retained 72.0%, indicating excellent flame-retardant properties [27]. The DPOSiDA/BPAF resin exhibited both the highest decomposition temperature and the lowest decomposition rate. These properties are attributed to the alkynyl structure, which enhances thermal resistance and suitability for extreme environments.

**Figure 3:**
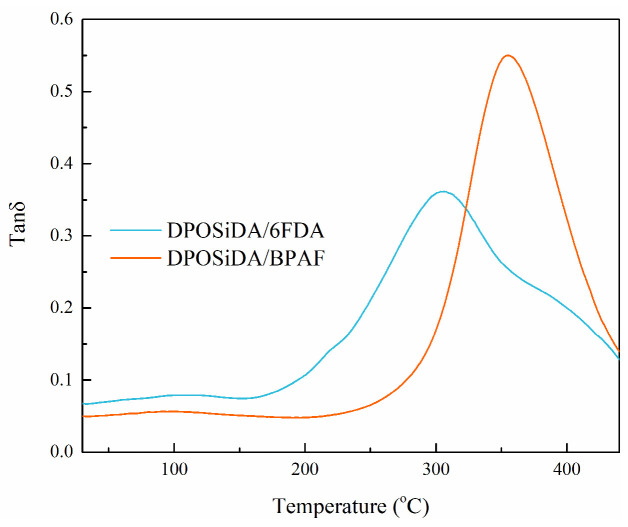
DMA curves of DPOSiDA/6FDA, and DPOSiDA/BPAF film.

To further assess the thermal properties, tanδ–T curves were recorded via DMA under identical testing conditions. As shown in Figure 3, both obtained polyimide films display a single tanδ peak corresponding to the glass-to-rubber transition [28]. The DPOSiDA/BPAF film shows a higher glass transition temperature (*T*_g_ = 354.6 °C), attributed to dense alkyne crosslinking and the rigid fluorenyl units of BPAF. Compared with 6FDA, BPAF introduces a rigid planar fluorene cardo-core and two ortho-phthalic anhydride substituents at the C-9 position, which increase steric hindrance, elevate rotational energy barriers, and suppress segmental mobility, collectively yielding a much higher *T*_g_. Upon copolymerization with BPAF, the rigid fluorene core and perfluorinated substituents synergistically increase segmental rigidity and reduce free volume, leading to decreased elongation at break and brittle films, which will be discussed in the subsequent section on DFT calculations.

Integration of the thermal analysis and temperature-resolved FT-IR spectra confirms that the superior thermal stability of the resin arises from crosslinking of the pendant alkyne functionalities. As illustrated in the curing section of Scheme 1, the internal alkyne units (phenylethynyl) undergo cyclotrimerization or Diels–Alder transformations during high-temperature curing, yielding a rigid, highly aromatic network enriched with benzene and naphthalene ring motifs. The resulting structural rigidity and high crosslinking density are key contributors to the resin’s enhanced thermal endurance.

### 3.3. Density Functional Theory (DFT) Analysis

**Figure 4:**
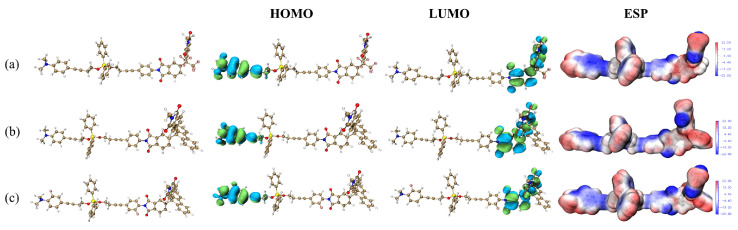
Optimized molecular configuration, molecular orbitals and ESP of DPOSiDA/6FDA (**a**), DPOSiDA/BPAF (**b**), and DPOSiFDA/BPAF (**c**). The polymer calculations were performed using the dimer of the repeating unit. To simplify the computation, methyl groups were used to replace the coupling between adjacent molecules.

Through DFT calculations on polymer fragments, the spatial configuration and charge electrostatic potential distribution of polymers DPOSiDA/6FDA, DPOSiDA/BPAF, and DPOSiFDA/BPAF were analyzed (Figure 4). All three polymers mainly exhibited twisted spatial configurations, with the bond angle around the silicon atom being approximately 103^o^. The dihedral angles between the fluorene-linked and phthalic anhydride-linked segments in compounds DPOSiDA/BPAF and DPOSiFDA/BPAF were about 89^o^, forming overall twisted conformations (The molecular structures from multiple viewing angles were provided in Figures S10–S12 and the full atomic coordinate data were listed in Tables S1–S3). Considering the higher planarity of the fluorene unit, it has a greater tendency to form planar stacking structures. Analysis of the HOMO and LUMO energy levels revealed that their distributions were mainly localized on the phthalic anhydride moiety, while the silicon atom primarily acted as a linkage site. The electrostatic potential analysis showed that the fluorine atoms on the DPOSiDA/6FDA substituents did not exhibit low potential regions, whereas those on the benzene ring displayed significantly lower electrostatic potentials, facilitating the formation of noncovalent interactions. Performance analysis indicated that the fluorene-modified molecules exhibited superior thermal stability, possibly because the planar structures within the twisted chain-like molecules can form spatially ordered π–π stacking interactions [29], which can help regulate polymer conformation in organic polymer systems. Electrostatic potential studies further revealed that the fluorine atoms on the benzene ring possess a lower electrostatic potential than those in trifluoromethyl groups, making the benzene-ring-substituted fluorine atoms more favorable for forming noncovalent interactions. DPOSiFDA/BPAF, in addition to π–π stacking, formed new noncovalent interactions, which disrupted the uniformity of molecular configuration. For polymers with planar structures, such additional noncovalent interactions increase rigidity but are unfavorable for improving film-forming performance [30]. In contrast, compound DPOSiDA/6FDA, lacking the structural regulation from fluorene, exhibited slightly inferior performance parameters due to its higher molecular freedom.

## 4. Discussion

The present work demonstrates that the incorporation of siloxy groups and fluorinated aromatic structures into polyimide backbones effectively regulates molecular packing, intermolecular interactions, and free volume, leading to improved solubility–thermal stability balance compared with conventional PI systems. Previous studies primarily relied on either bulky substituents or fluorine-containing dianhydrides to enhance solubility, but often at the expense of thermal resistance. Our results reveal that the dual-functional modification strategy synergistically mitigates this trade-off. The DFT calculations further elucidate the molecular mechanisms: twisted chain geometries weaken π–π stacking, while heterogeneous electrostatic potentials enable favorable noncovalent interactions (e.g., C–F···H–C), promoting solvent diffusion. Meanwhile, locally planar fluorene units retain inherent thermal stabilization benefits through constrained rotational mobility. To contextualize the performance of DPOSiDAs-PI, we compared our resins with representative silicon–alkyne polyimide resins. Notably, DPOSiDA/BPAF exhibits room-temperature solubility in DMAc/DMF/NMP/DMSO, together with a high *T*_d5%_ of 560 °C and a char yield of 72.0% at 800 °C under nitrogen, achieved after curing up to 300 °C. In comparison, a dual-functional silicon–alkyne polyimide (SiPI) featuring silylmethyl and alkyne moieties emphasizes improved processability (e.g., acetone/THF solubility), yet reports *T*_d5%_ = 511 °C and *R*_w800_ = 63% (N_2_) [31]. Likewise, a silicon-containing alkynyl polyimide resin (SiOPI) achieves *T*_d5%_ = 540.9 °C and *R*_w800_ = 65.76% (N_2_) with good processability [21], whereas DPOSiDA/BPAF provides a higher decomposition onset and higher high-temperature residue under identical inert-atmosphere metrics. These comparisons indicate that the present “spatially decoupled” siloxy–alkyne architecture provides an improved solubility-thermal stability balance, delivering a higher char yield and a higher Td5 than closely related silicon–alkyne designs while maintaining solution processability for fabrication of biomimetic and complex architectures.

These structure-property correlations provide guidance for designing next-generation soluble yet heat-resistant PI systems for advanced microelectronics and aerospace applications. However, further investigation is required to explore the influence of molecular weight distribution, side-group density, and processability in real device fabrication environments. Future studies should also involve quantitative simulations of chain mobility and dynamic cross-linking behavior at high service temperatures to expand high-performance application boundaries.

## 5. Conclusions

In summary, we developed a novel molecular design strategy to construct high-performance polyimides with a balanced combination of solubility and thermal resistance by integrating siloxyne and alkyne functionalities into diamine monomers as promising matrix candidates for biomimetic structural systems operating in extreme environments. The introduction of bulky siloxy groups effectively increases free volume and disrupts π–π stacking, leading to excellent solubility in common polar solvents. Meanwhile, the rigid fluorene core and alkyne crosslinking imparts outstanding thermal stability, with the DPOSiDA/BPAF resin exhibiting *T*_d5%_ up to 560 °C, *T*_g_ of 354.6 °C, and char yield of 72.0% at 800 °C. Complementary DFT calculations confirmed twisted molecular conformations and localized charge distributions that rationalize the amorphous packing and high thermal stability. Beyond establishing a predictive structure-property framework, this work establishes a materials platform highly suited for biomimetic structural systems intended for extreme environments, where matrix resins must provide exceptional thermal robustness, structural stability, and processing adaptability. The spatially decoupled siloxy–alkyne design offers a molecular strategy for tailoring PI-based matrices to meet the demands of emerging biomimetic architectures and other high-performance composites operating under severe thermal loads.

## Figures and Tables

**Table 1:** Solubilities of DPOSiDAs-PI resins in common organic solvents.

Sample	Solvent					
	DMAc	DMF	NMP	DMSO	THF	Toluene
DPOSiDA-6FDA	+	+	+	+	-	+
DPOSiDA-BPAF	++	++	++	++	-	+
DPOSiFDA-BPAF	++	++	++	++	-	+

++: Soluble; +: partially soluble; -: insoluble. The solubility was tested at 10 mg/mL.

**Table 2:** Thermal analysis data of DPOSiDAs-based polyimides.

Sample	N_2_				Air		
	*T*_d5%_ (°C)	*T*_d10%_ (°C)	*T*_max%_ (°C)	*R*_w800_ (%)	*T*_d5%_ (°C)	*T*_d10%_ (°C)	*T*_max%_ (°C)
DPOSiDA-6FDA	525 ± 0.6	542 ± 1	551 ± 1.1	56.0 ± 0.2	524 ± 1.5	540 ± 1	612 ± 2
DPOSiDA-BPAF	560 ± 1.1	602 ± 2	631 ± 1.1	72.0 ± 0.3	519 ± 1	546 ± 0.6	597 ± 0.6
DPOSiFDA-BPAF	541 ± 1	587 ± 1	610 ± 0.6	69.5 ± 0.1	506 ± 1.1	542 ± 2.6	601 ± 1

## Data Availability

The original contributions presented in this study are included in the article. Further inquiries can be directed to the corresponding author.

## Supplementary Materials

The following supporting information can be downloaded at: https://www.mdpi.com/article/10.3390/biomimetics11010061/s1, Figure S1: 1H NMR spectra of DPOSiDA; Figure S2: 13C NMR spectra of DPOSiDA; Figure S3: 1H NMR spectra of DPOSiFDA; Figure S4: 13C NMR spectra of DPOSiFDA; Figure S5: 19F NMR spectra of DPOSiFDA; Figure S6: Photographs of polyimide powders before curing (a–c) and after stepwise thermal curing (d–f): (a,d) DPOSiDA/6FDA; (b,e) DPOSiDA/BPAF; (c,f) DPOSiFDA/BPAF; Figure S7: FT-IR spectra of DPOSiDA/6FDA with different temperatures; Figure S8: FT-IR spectra of DPOSiDA/BPAF with different temperatures; Figure S9: FT-IR spectra of DPOSiFDA/BPAF with different temperatures; Figure S10: The molecular structures from multiple viewing angles of DPOSiDA/6FDA; Figure S11: The molecular structures from multiple viewing angles of DPOSiDA/BPAF; Figure S12: The molecular structures from multiple viewing angles of DPOSiFDA/BPAF; Table S1: Atomic coordinates of DPOSiDA/6FDA; Table S2: Atomic coordinates of DPOSiDA/BPAF; Table S3: Atomic coordinates of DPOSiFDA/BPAF.
