# Supplementary material for: High-Performance Polyimides with Enhanced Solubility and Thermal Stability for Biomimetic Structures in Extreme Environment
Source: Biomimetics (Basel). 2026 Jan 12;11(1):61. doi: 10.3390/biomimetics11010061 (PMC12839054; doi:10.3390/biomimetics11010061)
Supplement: Supplementary file 1 [file biomimetics-11-00061-s001.zip › biomimetics-4066501-supplementary.pdf]

High-Performance Polyimides with Enhanced Solubility and Thermal Stability for Biomimetic Structures in Extreme Environment

Jichao Chen[1], Jiping Yang[2], Zhiyong Ma[3], Zhijian Wang[1,2*], Yizhuo Gu[1,2*]

1 Tianmushan Laboratory, Hangzhou 310023, China; bhtb028@tmslab.cn

2 Key Laboratory of Aerospace Advanced Materials and Performance, Ministry of Education, School of

Materials Science and Engineering, Beihang University, Beijing 100191, China

3 Beijing Advanced Innovation Center for Soft Matter Science and Engineering, State Key Laboratory of

Organic-Inorganic Composites, College of Chemical Engineering, Beijing University of Chemical Technology,

Beijing 100029, China

* Correspondence: zhijianw@buaa.edu.cn (Z.W.); benniegu@buaa.edu.cn (Y.G.)

Table of Contents

1. ^{1}H, ^{13}C and ^{19}F NMR spectra S1

2 Photographs of polyimide powders S3

3. FT-IR spectra S4

4 The molecular structures from multiple viewing angles of DPOSiDA/6FDA, DPOSiDA/BPAF, and DPOSiFDA/BPAF S6

5 The full atomic coordinate data of DPOSiDA/6FDA, DPOSiDA/BPAF, and DPOSiFDA/BPAF S7

1. ^{1}H, ^{13}C and ^{19}F spectra

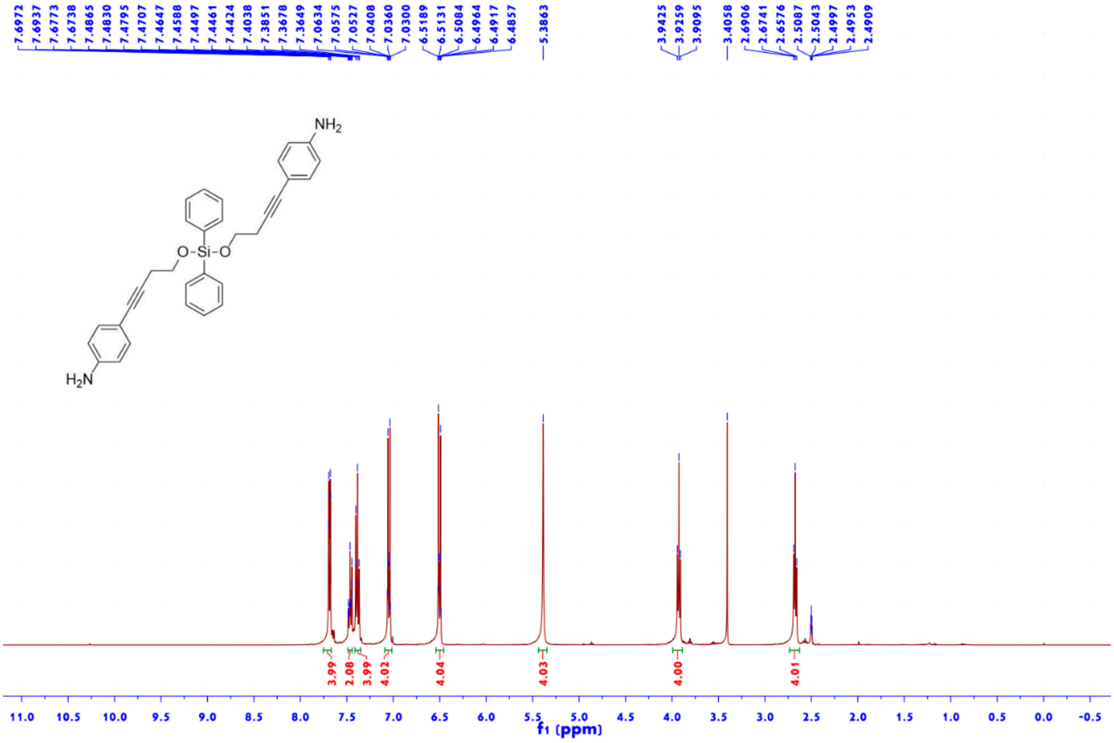

Figure S1. ^{1}H NMR spectra of DPOSiDA.

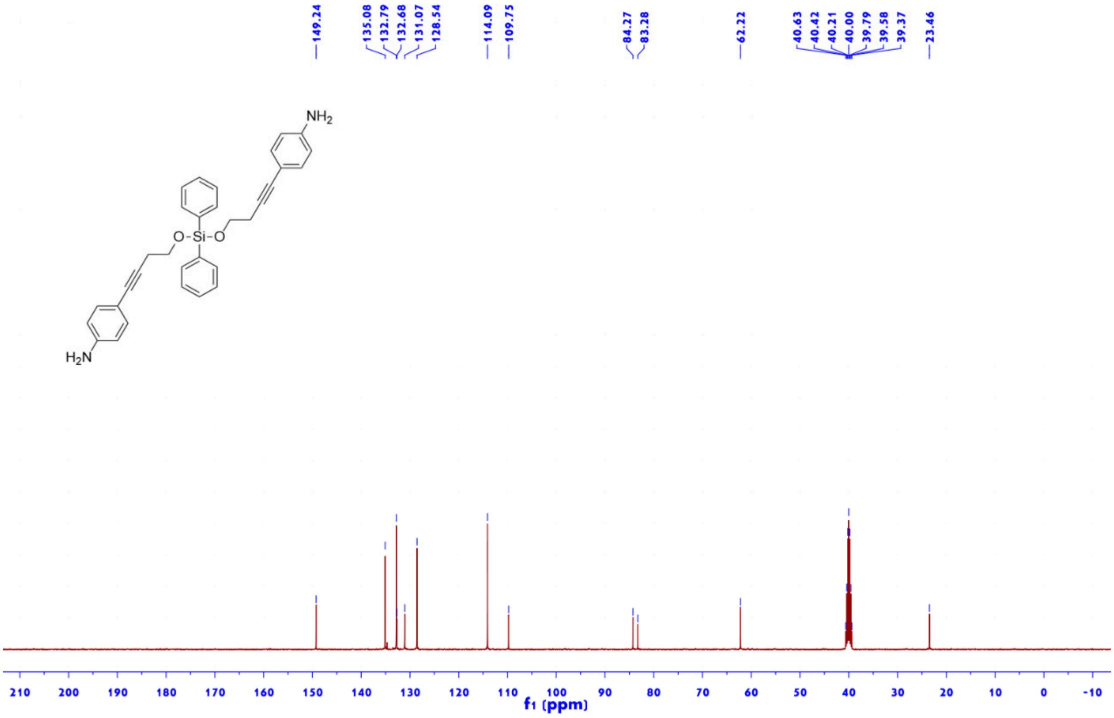

Figure S2. ^{13}C NMR spectra of DPOSiDA.

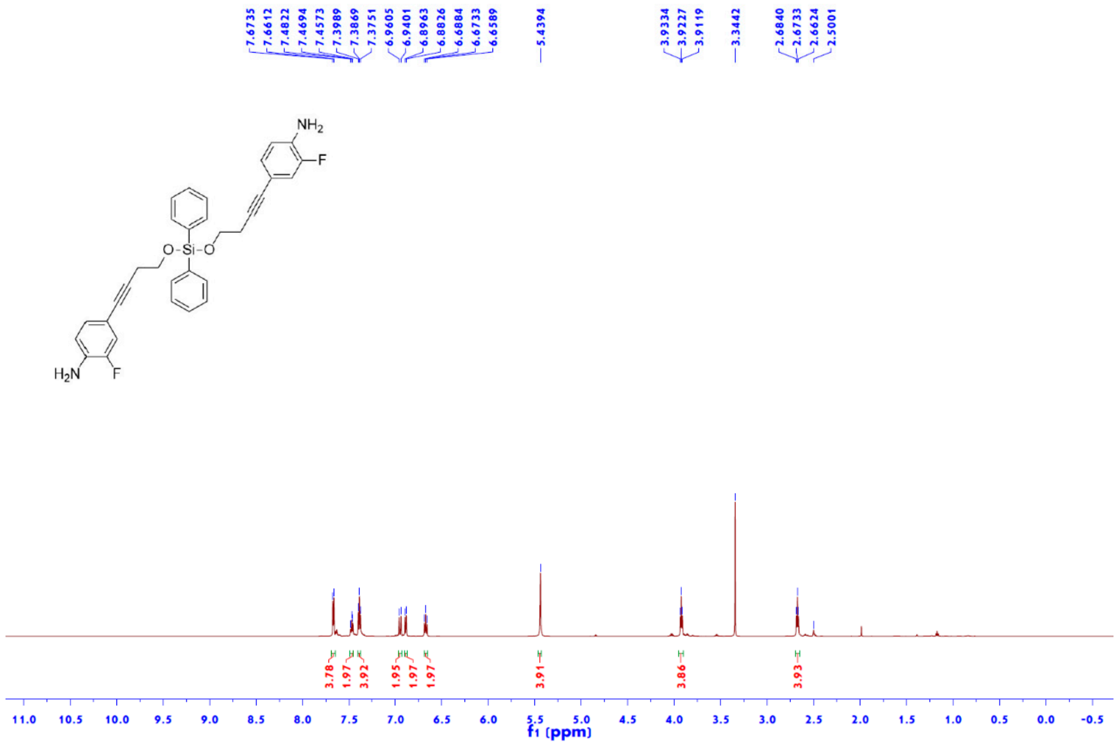

Figure S3. ^{1}H NMR spectra of DPOSiFDA.

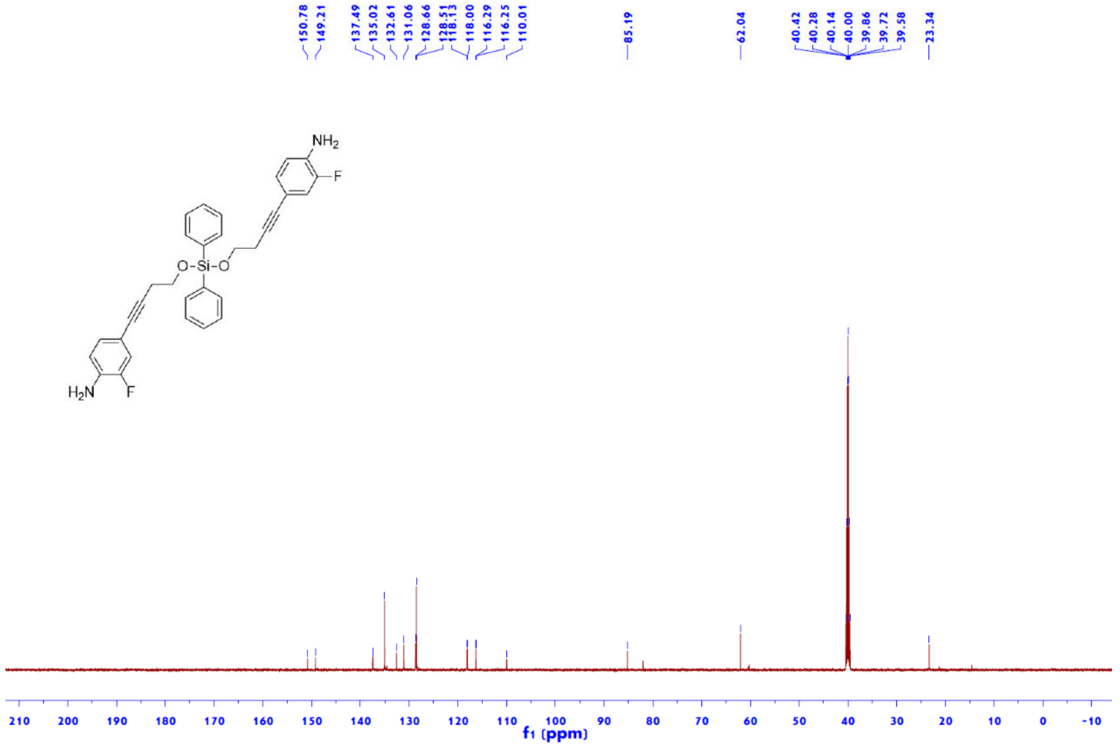

Figure S4. ^{13}C NMR spectra of DPOSiFDA.

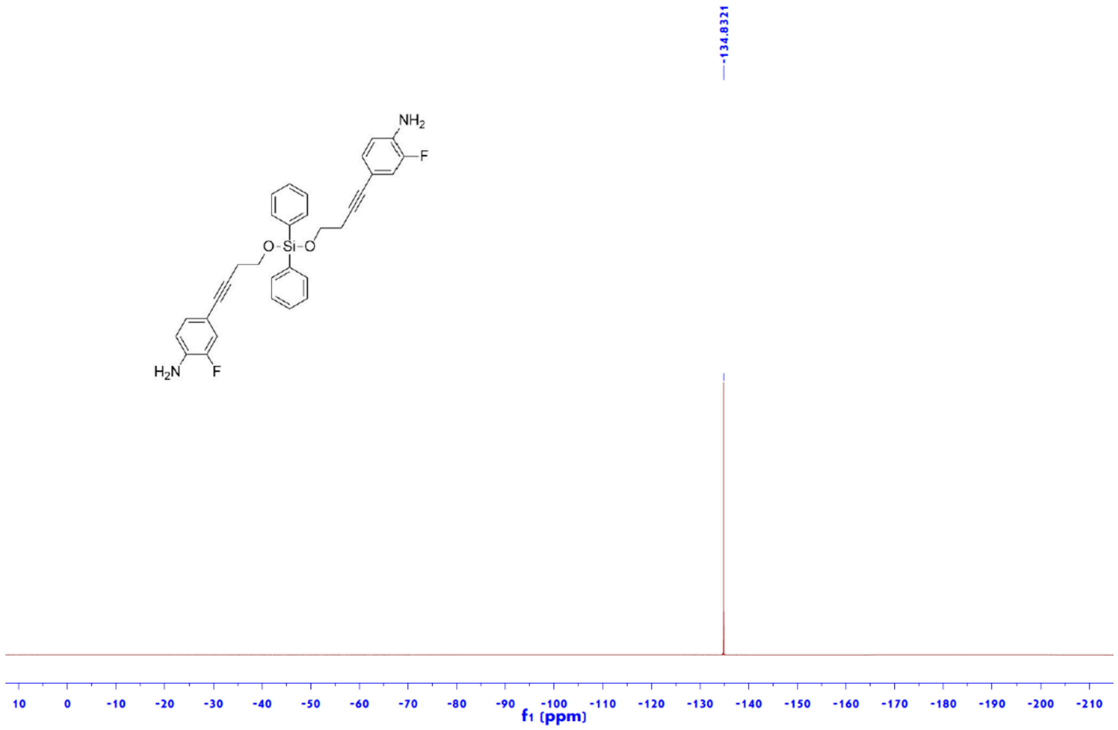

Figure S5. ^{19}F NMR spectra of DPOSiFDA.

2. Photographs of polyimide powders

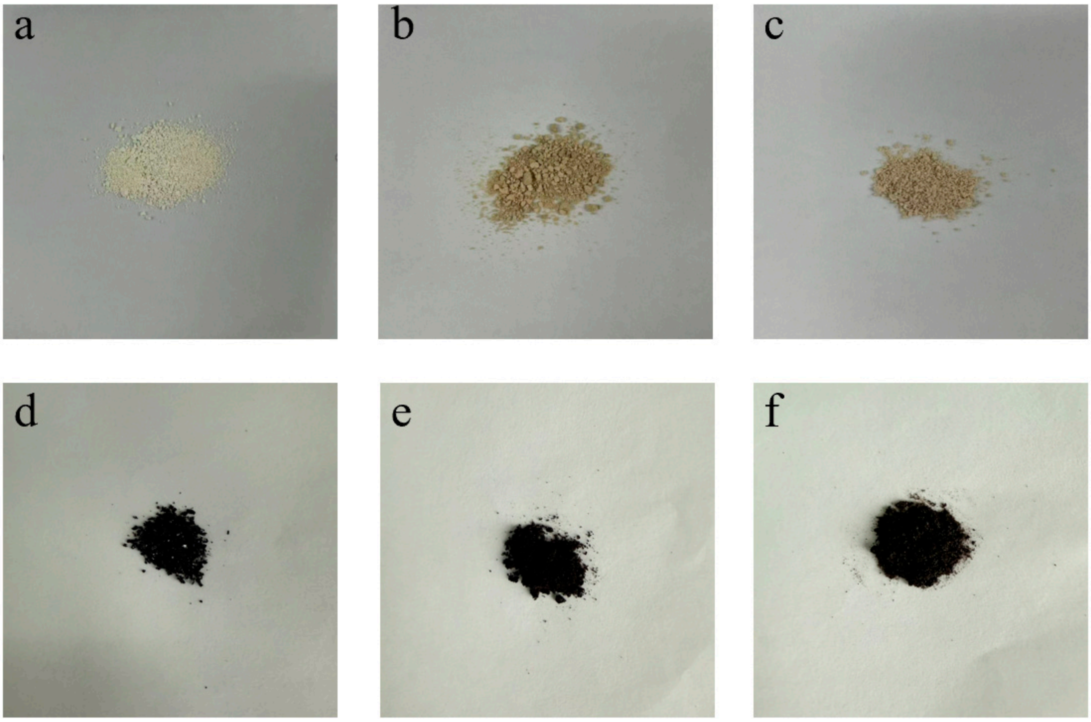

Figure S6. Photographs of polyimide powders before curing (a-c) and after stepwise thermal curing (d-f): (a,d) DPOSiDA/6FDA; (b,e) DPOSiDA/BPAF; (c,f) DPOSiFDA/BPAF.

3. FT-IR spectra

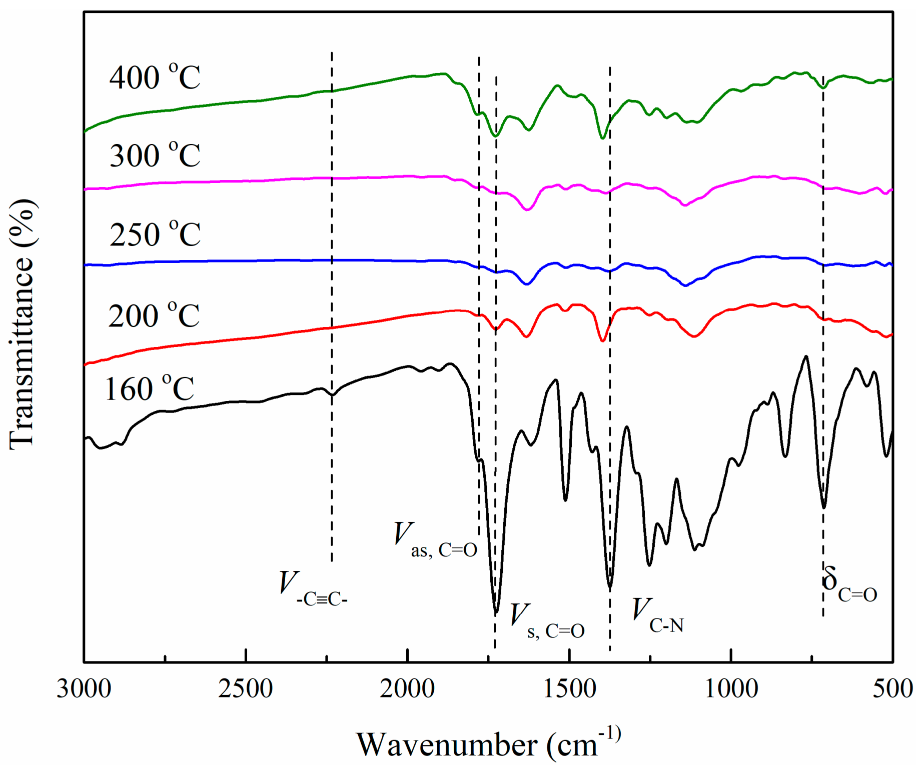

Figure S7. FT-IR spectra of DPOSiDA/6FDA with different temperatures.

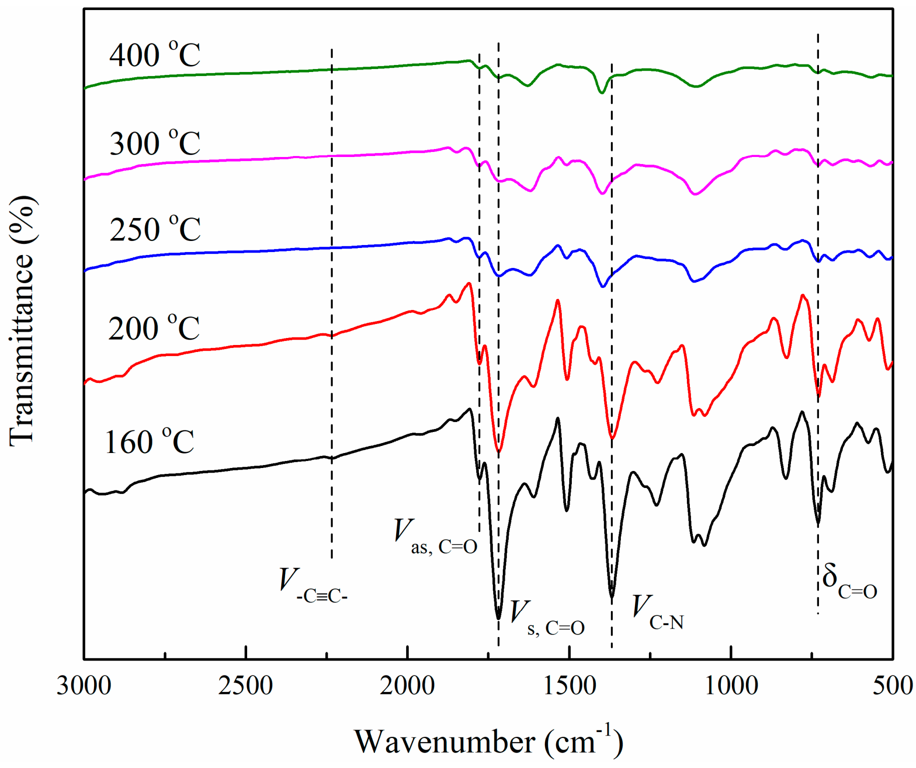

Figure S8. FT-IR spectra of DPOSiDA/BPAF with different temperatures.

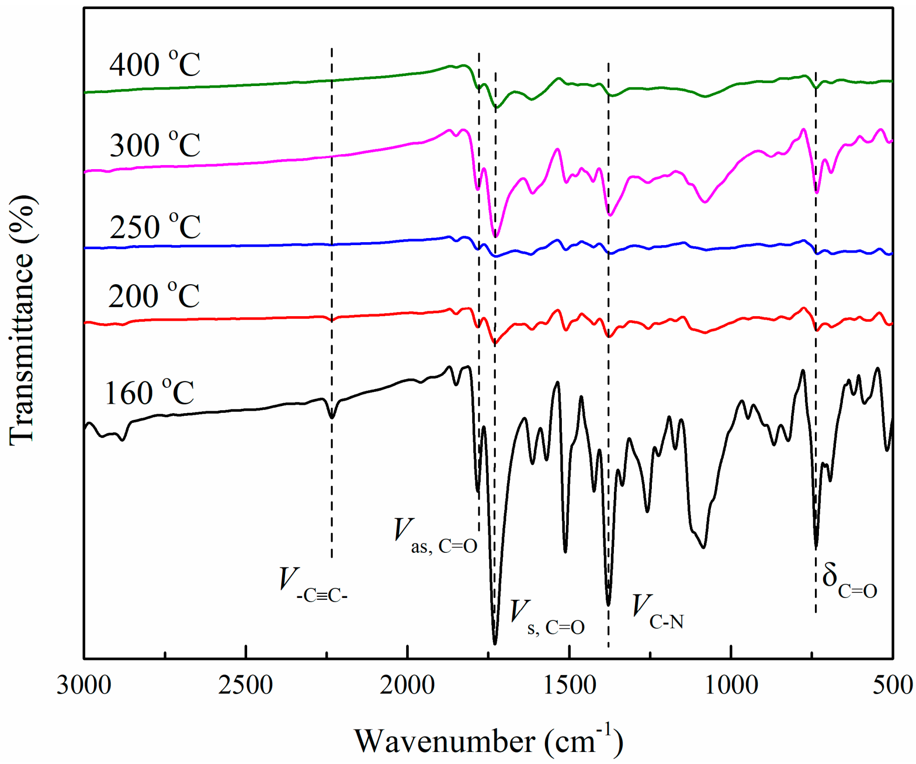

Figure S9. FT-IR spectra of DPOSiFDA/BPAF with different temperatures.

4. The molecular structures from multiple viewing angles of DPOSiDA/6FDA, DPOSiDA/BPAF, and DPOSiFDA/BPAF

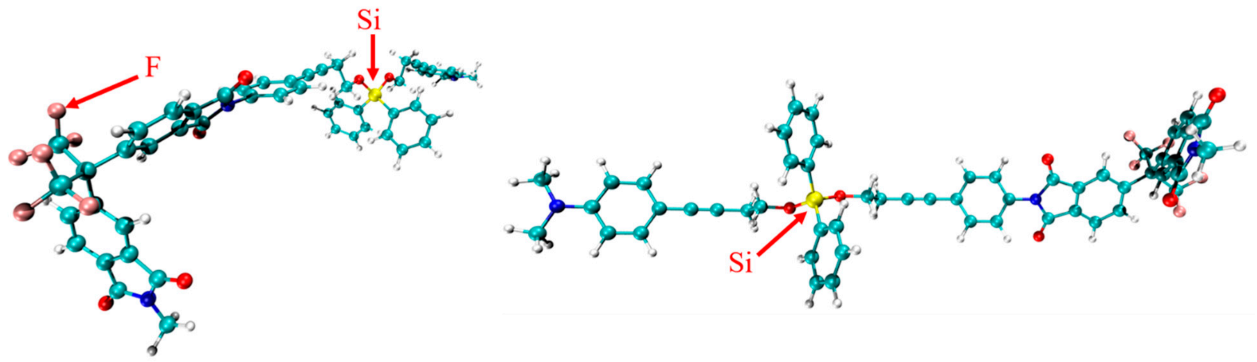

Figure S10. The molecular structures from multiple viewing angles of DPOSiDA/6FDA.

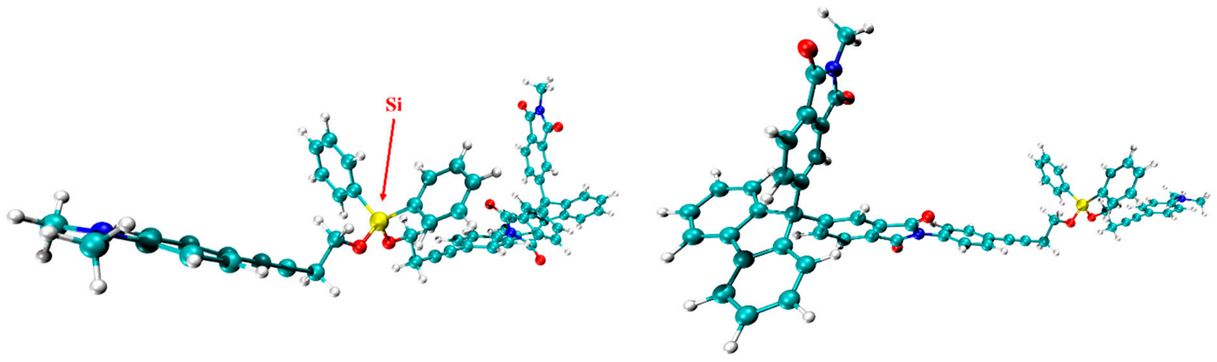

Figure S11. The molecular structures from multiple viewing angles of DPOSiDA/BPAF.

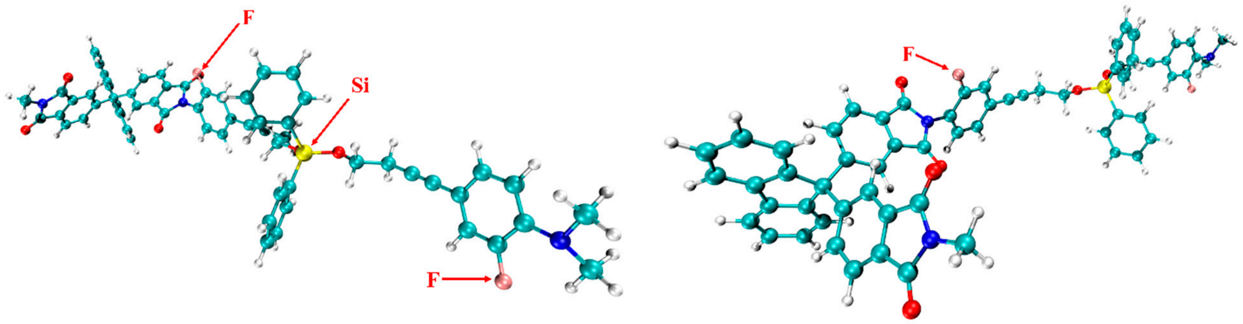

Figure S12. The molecular structures from multiple viewing angles of DPOSiFDA/BPAF.

5. The full atomic coordinate data of DPOSiDA/6FDA, DPOSiDA/BPAF, and DPOSiFDA/BPAF

Table S1. Atomic coordinates of DPOSiDA/6FDA.

Atom	x	y	z
Si	-6.76236	-0.14942	0.025025
O	-8.14575	-1.06607	0.034669
O	-5.62276	-1.24737	-0.48508
C	-9.47635	-0.55849	0.036548
C	-4.21791	-1.12061	-0.31423
C	-3.57722	-2.48218	-0.62485
C	-2.12219	-2.454	-0.4983
C	-0.916	-2.39545	-0.38325
C	-10.4348	-1.72476	-0.24304
C	-11.8353	-1.30753	-0.23991
C	-12.9874	-0.92555	-0.22985
C	0.504873	-2.33978	-0.25029
C	-14.3464	-0.48923	-0.22218
C	-14.9671	-0.0069	-1.3894
C	-16.2885	0.416458	-1.38852
C	-17.0653	0.388118	-0.20648
C	-16.4444	-0.10887	0.963568
C	-15.1222	-0.52959	0.951136
C	1.117158	-2.48088	1.009256
C	2.498835	-2.41918	1.143652
C	3.301326	-2.22748	0.012318
C	2.710431	-2.09206	-1.24989
C	1.327398	-2.14114	-1.3751
C	9.329947	-1.44288	0.091757
C	10.66189	-0.81298	-0.3822
C	8.138984	-0.98097	-0.50065
C	6.946255	-1.56973	-0.1193
C	6.899617	-2.59117	0.823575
C	8.058231	-3.06959	1.411527
C	9.274035	-2.4904	1.028128
C	10.54389	0.727141	-0.48007
C	11.0843	1.465627	-1.54671
C	10.99825	2.863114	-1.59141
C	10.37631	3.508826	-0.53726
C	9.853604	2.786475	0.534366
C	9.923779	1.406245	0.587066

C	10.13054	4.962479	-0.28658
C	9.257182	3.751976	1.507515
C	5.557201	-1.27772	-0.57445
N	4.72083	-2.16987	0.145529
C	5.478162	-2.9969	1.015851
O	8.705609	3.525877	2.565008
O	10.42714	5.920086	-0.97105
O	5.196071	-0.45579	-1.38872
O	5.038995	-3.85049	1.755527
N	-18.3785	0.837563	-0.19316
C	-6.85473	1.260709	-1.20918
C	-7.39159	2.519045	-0.87868
C	-7.48349	3.537716	-1.82829
C	-7.03476	3.318085	-3.13165
C	-6.49603	2.078561	-3.48185
C	-6.40889	1.0626	-2.52987
C	-6.41215	0.465847	1.762808
C	-5.58445	1.574802	2.019847
C	-5.31096	1.985537	3.325426
C	-5.86551	1.294453	4.403983
C	-6.692	0.19309	4.173161
C	-6.96017	-0.21528	2.86649
H	-9.71437	-0.11079	1.010457
H	-9.60163	0.216385	-0.72975
H	-3.81413	-0.35937	-0.99494
H	-3.96992	-0.82023	0.711415
H	-4.00584	-3.22972	0.053862
H	-3.86638	-2.77967	-1.6403
H	-10.1672	-2.16801	-1.21037
H	-10.2644	-2.50264	0.51195
H	-14.3977	0.02906	-2.31292
H	-16.9968	-0.16983	1.892852
H	-14.6748	-0.90435	1.866473
H	0.496903	-2.634	1.885799
H	0.870788	-2.0315	-2.35285
H	8.142141	-0.18556	-1.23605
H	8.026709	-3.87024	2.142633
H	10.18281	-2.87051	1.473281
H	11.5829	0.959339	-2.36133
H	11.41129	3.420389	-2.42544
H	9.507054	0.87253	1.432744
H	-7.73541	2.710935	0.134853

H	-7.90203	4.501324	-1.55163
H	-7.10318	4.11069	-3.87152
H	-6.14405	1.905068	-4.49495
H	-5.98952	0.100296	-2.8112
H	-5.15406	2.131831	1.191099
H	-4.66901	2.844228	3.501008
H	-5.65587	1.614292	5.420876
H	-7.12725	-0.34533	5.010425
H	-7.6042	-1.07375	2.695458
C	-19.2018	0.604175	0.980888
H	-18.7699	1.083401	1.866769
H	-20.1871	1.043608	0.819414
H	-19.3352	-0.46547	1.205755
C	-19.0409	1.146528	-1.44877
H	-18.5149	1.943773	-1.98615
H	-19.1146	0.277054	-2.1204
H	-20.0508	1.503273	-1.24163
N	9.463151	5.02239	0.945407
C	9.03374	6.263171	1.565928
H	8.54654	6.00737	2.507071
H	9.892861	6.91178	1.755429
H	8.332405	6.791716	0.915228
H	3.329494	-1.93511	-2.12354
C	11.84523	-1.09638	0.593336
C	10.99448	-1.48233	-1.75102
F	12.20639	-2.39765	0.609444
F	12.93281	-0.3874	0.254403
F	11.51426	-0.75484	1.854886
F	10.90774	-2.81908	-1.67042
F	12.23904	-1.18869	-2.18486
F	10.12962	-1.07602	-2.70211
H	2.955106	-2.53308	2.118317
H	-16.7176	0.770627	-2.31738

Table S2. Atomic coordinates of DPOSiDA/BPAF.

Atom	x	y	z
Si	-7.23774	-0.15476	0.06334
O	-8.60757	-1.09053	0.011515
O	-6.07562	-1.21025	-0.4832
C	-9.94515	-0.60214	0.020433
C	-4.67446	-1.06737	-0.2918

C	-4.00487	-2.3966	-0.67267
C	-2.55192	-2.34836	-0.52872
C	-1.34835	-2.27325	-0.39681
C	-10.8832	-1.7668	-0.3271
C	-12.2895	-1.36956	-0.32229
C	-13.4469	-1.00404	-0.30895
C	0.069772	-2.19771	-0.24397
C	-14.8118	-0.58671	-0.29826
C	-15.4279	-0.06835	-1.45238
C	-16.755	0.336992	-1.44861
C	-17.5426	0.25317	-0.27637
C	-16.9261	-0.28006	0.879968
C	-15.5982	-0.68238	0.864763
C	0.670071	-2.3815	1.015735
C	2.048603	-2.30016	1.170172
C	2.862023	-2.04552	0.058673
C	2.282265	-1.86707	-1.20383
C	0.901951	-1.9359	-1.34842
C	8.878448	-1.16221	0.238849
C	10.23153	-0.5618	-0.19744
C	7.684685	-0.70395	-0.35258
C	6.496422	-1.32378	-0.00426
C	6.453655	-2.37338	0.908863
C	7.613686	-2.84583	1.502125
C	8.82094	-2.22892	1.153973
C	10.15526	0.967586	-0.38979
C	11.38591	-0.86525	0.778618
C	12.36377	-1.66717	0.160995
C	11.93895	-1.95839	-1.2128
C	10.70723	-1.32107	-1.45225
C	12.54878	-2.7201	-2.21166
C	11.9167	-2.83875	-3.45046
C	10.69118	-2.20761	-3.68689
C	10.07715	-1.44732	-2.6857
C	11.55233	-0.43819	2.09174
C	12.70012	-0.82619	2.79141
C	13.67093	-1.62526	2.178942
C	13.51074	-2.05037	0.859156
C	10.86221	1.604247	-1.42458
C	10.87608	2.997365	-1.56818
C	10.16609	3.749318	-0.64679
C	9.464556	3.13104	0.388585

C	9.442525	1.755209	0.536721
C	9.978922	5.225296	-0.52352
C	8.808923	4.192277	1.211553
C	5.109832	-1.0436	-0.4716
N	4.277253	-1.96751	0.212582
C	5.039749	-2.80679	1.070689
O	8.116325	4.072014	2.20235
O	10.41805	6.119299	-1.21874
O	4.745472	-0.20882	-1.27249
O	4.601512	-3.68808	1.778878
N	-18.8618	0.684683	-0.25903
C	-7.33649	1.31103	-1.10391
C	-7.89563	2.544558	-0.72103
C	-7.99091	3.605912	-1.62234
C	-7.52324	3.454896	-2.9288
C	-6.96226	2.241117	-3.33045
C	-6.87185	1.182297	-2.4267
C	-6.91916	0.382965	1.832688
C	-6.11024	1.489469	2.152362
C	-5.85908	1.842029	3.479252
C	-6.41773	1.093803	4.516791
C	-7.22586	-0.00635	4.223669
C	-7.47165	-0.35645	2.895857
H	-10.2018	-0.20513	1.011468
H	-10.0723	0.206865	-0.70944
H	-4.27946	-0.26113	-0.92411
H	-4.4417	-0.82121	0.751667
H	-4.42603	-3.18956	-0.0425
H	-4.27853	-2.64079	-1.70652
H	-10.5973	-2.15933	-1.31097
H	-10.7108	-2.57762	0.391951
H	-14.8503	0.010609	-2.36807
H	-17.4865	-0.38396	1.800657
H	-15.1545	-1.08597	1.769565
H	0.04263	-2.58328	1.877264
H	2.495295	-2.44776	2.14457
H	0.455098	-1.79239	-2.32638
H	7.680225	0.120218	-1.05737
H	7.584785	-3.66456	2.213475
H	9.740289	-2.58275	1.605868
H	13.49862	-3.2148	-2.03065
H	12.37882	-3.42827	-4.23661

H	10.20888	-2.31056	-4.65412
H	9.121921	-0.96817	-2.87768
H	10.80933	0.189169	2.574643
H	12.83854	-0.50006	3.817687
H	14.55793	-1.91513	2.734247
H	14.26852	-2.66788	0.38571
H	11.41266	0.998423	-2.13495
H	11.42364	3.472739	-2.37541
H	8.876315	1.306004	1.345373
H	-8.25425	2.683163	0.296048
H	-8.42667	4.549344	-1.30563
H	-7.59414	4.280839	-3.63102
H	-6.59535	2.121054	-4.346
H	-6.43494	0.240494	-2.74788
H	-5.67692	2.090231	1.356355
H	-5.23115	2.699786	3.703357
H	-6.22536	1.368205	5.550243
H	-7.66411	-0.58932	5.028938
H	-8.10117	-1.21464	2.676297
C	-19.6932	0.394507	0.896526
H	-19.2761	0.843921	1.804811
H	-20.6826	0.827405	0.742571
H	-19.8147	-0.68481	1.077862
C	-19.5167	1.030869	-1.50886
H	-18.9966	1.85418	-2.01144
H	-19.5725	0.185833	-2.21266
H	-20.5332	1.366556	-1.29856
N	9.161295	5.407776	0.603128
C	8.733629	6.709236	1.082442
H	8.11369	6.545933	1.964367
H	9.599018	7.323729	1.344413
H	8.15634	7.228782	0.313169
H	2.908325	-1.66039	-2.0618
H	-17.1801	0.720717	-2.36754

Table S3. Atomic coordinates of DPOSiFDA/BPAF.

Atom	x	y	z
Si	6.986587	-0.11946	-0.02548
O	8.353573	-1.04795	-0.19256
O	5.841923	-1.26056	0.364696
C	9.690745	-0.56288	-0.16584

C	4.43611	-1.09793	0.239807
C	3.785774	-2.48474	0.36312
C	2.32957	-2.4258	0.266558
C	1.122913	-2.34036	0.179658
C	10.63343	-1.76926	-0.04431
C	12.04069	-1.37681	-0.0335
C	13.19878	-1.01491	-0.0282
C	-0.29893	-2.25263	0.081566
C	14.56557	-0.60386	-0.02162
C	15.19611	-0.21408	1.176635
C	16.51451	0.192882	1.164962
C	17.31045	0.217832	-0.00073
C	16.6597	-0.17356	-1.1868
C	15.3244	-0.56624	-1.20267
C	-0.91541	-2.08816	-1.1722
C	-2.29489	-1.99535	-1.24807
C	-3.10642	-2.08857	-0.11238
C	-2.49031	-2.24836	1.13256
C	-1.1063	-2.32252	1.233673
C	-9.0975	-1.12993	-0.20611
C	-10.4368	-0.51077	0.246224
C	-7.89142	-0.69598	0.37801
C	-6.71552	-1.33055	0.013852
C	-6.69777	-2.37625	-0.90745
C	-7.87182	-2.82612	-1.4906
C	-9.06546	-2.1912	-1.12861
C	-10.3337	1.015398	0.451657
C	-11.6037	-0.78594	-0.72334
C	-12.5907	-1.576	-0.10485
C	-12.1603	-1.88653	1.262983
C	-10.9158	-1.2729	1.498018
C	-12.7755	-2.64625	2.260103
C	-12.1358	-2.78678	3.492709
C	-10.8976	-2.17922	3.724701
C	-10.2783	-1.421	2.72519
C	-11.7726	-0.34511	-2.03158
C	-12.9323	-0.70721	-2.72547
C	-13.9123	-1.49431	-2.11209
C	-13.7495	-1.93326	-0.79717
C	-11.0212	1.653946	1.498364
C	-11.0111	3.04571	1.654693
C	-10.2971	3.794477	0.733876

C	-9.61476	3.17446	-0.31325
C	-9.61627	1.799829	-0.47398
C	-10.0873	5.268321	0.622363
C	-8.9495	4.232647	-1.13248
C	-5.32054	-1.07495	0.473729
N	-4.5159	-2.00114	-0.2351
C	-5.29356	-2.84132	-1.07798
O	-8.26777	4.110762	-2.1305
O	-10.5059	6.162768	1.329693
O	-4.92355	-0.26635	1.284691
O	-4.86534	-3.74774	-1.75543
N	18.638	0.672615	0.036574
C	7.136551	1.111003	1.382133
C	7.69296	2.392119	1.208303
C	7.82774	3.273218	2.282248
C	7.403317	2.889991	3.555588
C	6.845774	1.625025	3.751354
C	6.715696	0.74676	2.675388
C	6.60573	0.724867	-1.6567
C	5.796304	1.873172	-1.74202
C	5.497508	2.456846	-2.9741
C	6.007825	1.903293	-4.1495
C	6.81553	0.766025	-4.0891
C	7.109116	0.184553	-2.85554
H	9.916757	-0.00937	-1.08681
H	9.846447	0.115912	0.681793
H	4.053089	-0.43937	1.030593
H	4.173861	-0.65069	-0.72702
H	4.19457	-3.13063	-0.42357
H	4.087016	-2.92781	1.320169
H	10.3803	-2.31588	0.872537
H	10.43345	-2.45234	-0.87923
H	14.65448	-0.20324	2.115403
H	17.21397	-0.18141	-2.11753
H	14.86311	-0.86188	-2.13887
H	-0.32865	-2.01924	-2.08028
H	-0.63751	-2.44377	2.203461
H	-7.86791	0.122201	1.089413
H	-7.8634	-3.6416	-2.20621
H	-9.99435	-2.52687	-1.57485
H	-13.7352	-3.1227	2.082463
H	-12.602	-3.37499	4.277444

H	-10.4096	-2.29901	4.687145
H	-9.31334	-0.96015	2.913455
H	-11.0224	0.273	-2.51519
H	-13.0727	-0.37028	-3.74798
H	-14.8084	-1.76403	-2.66289
H	-14.5143	-2.54152	-0.32298
H	-11.5753	1.050698	2.20806
H	-11.5439	3.522474	2.470976
H	-9.0644	1.348907	-1.29155
H	8.017882	2.7113	0.220951
H	8.260463	4.25732	2.125811
H	7.505135	3.575499	4.392211
H	6.512478	1.324739	4.740824
H	6.281702	-0.23647	2.836527
H	5.400318	2.324153	-0.83533
H	4.870169	3.342722	-3.01708
H	5.778181	2.357642	-5.10922
H	7.216052	0.334237	-5.00203
H	7.737965	-0.70098	-2.8175
C	19.27822	0.960632	-1.23597
H	18.63362	1.596105	-1.8484
H	20.20655	1.505519	-1.04182
H	19.53472	0.057883	-1.81785
C	19.54893	0.045244	0.996249
H	19.06534	-0.06583	1.963695
H	19.89188	-0.94559	0.654464
H	20.42758	0.685089	1.123469
N	-9.27696	5.448068	-0.50981
C	-8.83293	6.746818	-0.98145
H	-8.22335	6.58158	-1.87021
H	-9.6907	7.377211	-1.23042
H	-8.24083	7.250363	-0.21278
H	-3.10927	-2.3051	2.020271
F	-2.86988	-1.79509	-2.44644
F	17.056	0.601683	2.341215
